# Chaperone Sigma1R mediates the neuroprotective action of afobazole in the 6-OHDA model of Parkinson’s disease

**DOI:** 10.1038/s41598-019-53413-w

**Published:** 2019-11-19

**Authors:** Mikhail V. Voronin, Ilya A. Kadnikov, Dmitry N. Voronkov, Sergey B. Seredenin

**Affiliations:** 1 Federal State Budgetary Institution “Research Zakusov Institute of Pharmacology”, Department of Pharmacogenetics, Baltiyskaya street 8, Moscow, 125315 Russian Federation; 2 Research Center of Neurology, Laboratory of Functional Morphochemistry, Volokolamskoe Highway 80, Moscow, 125367 Russian Federation

**Keywords:** Target validation, Behavioural methods, Liquid chromatography, Regeneration and repair in the nervous system, Animal disease models

## Abstract

Parkinson’s disease (PD) is a progressive neurodegenerative disease with limited treatment options. Therefore, the identification of therapeutic targets is urgently needed. Previous studies have shown that the ligand activation of the sigma-1 chaperone (Sigma1R) promotes neuroprotection. The multitarget drug afobazole (5-ethoxy-2-[2-(morpholino)-ethylthio]benzimidazole dihydrochloride) was shown to interact with Sigma1Rs and prevent decreases in striatal dopamine in the 6-hydroxydopamine (6-OHDA)-induced parkinsonism model. The aim of the present study was to elucidate the role of Sigma1Rs in afobazole pharmacological activity. Using ICR mice we found that administration of afobazole (2.5 mg/kg, i.p.) or selective agonist of Sigma1R PRE-084 (1.0 mg/kg, i.p.) over 14 days normalizes motor disfunction and prevents decreases in dopamine in the 6-OHDA-lesioned striatum. Afobazole administration also prevents the loss of TH + neurons in the substantia nigra. The pre-administration of selective Sigma1R antagonist BD-1047 (3.0 mg/kg, i.p.) abolishes the activity of either afobazole or PRE-084, as determined using the rotarod test and the analysis of striatal dopamine content. The current study demonstrates the contribution of Sigma1Rs in the neuroprotective effect of afobazole in the 6-OHDA model of Parkinson’s disease and defines the therapeutic perspective of Sigma1R agonists in the clinic.

## Introduction

Parkinson’s disease is a neurodegenerative condition caused by injury in dopamine-producing regions of the brain, especially in neurons of the substantia nigra pars compacta (SNc) and their axonal projections to the striatum. Parkinson’s disease is characterized by progressive movement impairments including bradykinesia, resting tremor, rigidity and postural instability^1^. Epidemiological data revealed a two-fold increase in the prevalence of this disease over the last 25 years, outranked only by Alzheimer’s disease among all neurodegenerative diseases^2^. Despite several studies suggesting the role of environmental and genetic factors, the etiology of Parkinson’s disease is still poorly understood^3^. The main pathogenetic components of the disease are mitochondrial damage, impaired protein proteasomal degradation, misfolding, oxidative stress and neuroinflammation^1,4–6^. Levodopa, MAO-B and COMT inhibitors, dopamine agonists, β-blockers, and adamantane derivatives with NMDA receptor antagonism are the most commonly used treatment options^7,8^. The mechanism of action of these drugs involves the facilitation of dopaminergic transmission in compromised neurons or the correction of imbalance in neuromediator systems.

The Sigma-1 receptor (Sigma1R)^9^ is a promising target for the development of therapy for Parkinson’s disease^10–13^. The expression and cellular localization of Sigma1Rs in the brain have been described in previous studies^12,14^. The nigrostriatal motor system has a significant level of Sigma1Rs^15,16^. The most important functions of Sigma1Rs include the ability for ligand activation, intracellular translocation and chaperone activity towards target proteins^14,17,18^. Various damaging factors lead to the accumulation of aberrant proteins, which activate the unfolded protein response (UPR) or endoplasmic-reticulum-associated protein degradation (ERAD)^19^. In the adaptive stage of endoplasmic reticulum (ER) stress and in response to ligand activation, Sigma1Rs dissociate from the main ER chaperone binding-immunoglobulin protein (BiP, GRP-78), which facilitates proper protein folding. Sigma1R also performs chaperone functions by stabilizing the ER stress sensor serine/threonine-protein kinase/endoribonuclease IRE1 (IRE1a) and inositol 1,4,5-trisphosphate receptor type 3 (IP3R3). These protein-protein interactions enhance the expression of ER chaperone genes, maintaining the required Ca^2+^ current in mitochondria, reducing ROS production and preventing the apoptotic transformation of neurons^14,20–23^. Sigma1Rs are able to bind specifically a variety of ligands^17,24^. In response to ligand activation, the Sigma1R enters a monomeric state and, as a part of the lipid domain, translocates to the region of nuclear and cytoplasmic membranes, where it regulates the activity of different receptors, ion channels and enzymes^18,25^. This activity prevents excitotoxicity and glial activation and may promote neuroprotective effects in Parkinson’s disease^26–34^. The contribution of Sigma1Rs in the attenuation of dopamine and glutamate toxicity was previously defined using *in vitro* models^22,35^. However, studies of the effects of the Sigma1R selective agonist PRE-084 in MPTP and 6-OHDA models of Parkinson’s disease produced contradictory results^11,36^.

The anxiolytic drug afobazole (ethoxy-2-[2-(morpholino)-ethylthio]benzimidazole dihydrochloride) was developed and pharmacologically studied at the FSBI “Research State Zakusov Institute of Pharmacology”^37^. The drug has affinity to Sigma1Rs (Ki = 5.9E-6 M) and regulatory sites of NQO2 (Ki = 9.7E-7 M) and MAO A (Ki = 3.6E-06 M)^38^. *In vitro* and *in vivo* experiments with afobazole revealed cytoprotective and neuroprotective properties of the drug^39–42^. Our recent results showed the ability of afobazole to prevent decreases in striatal dopamine in the model of 6-OHDA-induced parkinsonism^43^; however, the role of the above mentioned molecular targets of afobazole has not been defined. The aim of the present study was to determine the role of Sigma1Rs in the afobazole-mediated normalizing effect on dopamine content in the striatum in an *in vivo* mouse model of Parkinson’s disease induced by 6-OHDA lesions.

## Results

**Figure 1:**
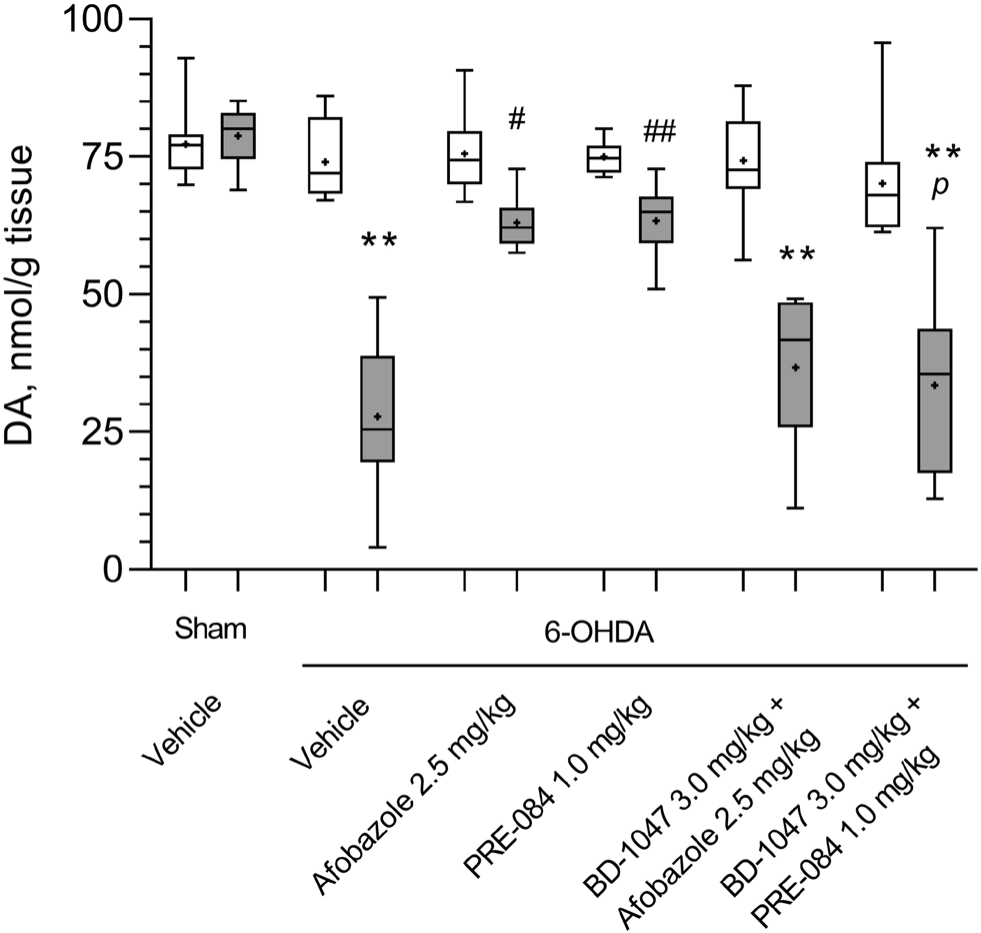
The influence of Sigma1R ligand administration over 14 days on dopamine content in the intact and 6-OHDA lesioned striatum of ICR mice. Data are presented as the Mdn (min-max). “+” – the mean. Sham – sham-operated mice. 6-OHDA – 6-OHDA-lesioned mice. Experimental groups consisted of 10 mice except for afobazole-treated only, which was 9. A significant difference between the contra- and ipsilateral striata was observed in all experimental groups with 6-OHDA lesions (p < 0.01, Wilcoxon test).  -contralateral striatum,  - ipsilateral striatum. **p < 0.01- statistical significance versus lesioned striatum of sham-operated mice (Kruskal–Wallis test, Dunn’s post hoc test). ^#^p < 0.05, ^##^p < 0.01- statistical significance versus 6-OHDA lesioned striatum of vehicle-treated mice (Kruskal–Wallis test, Dunn’s post hoc test). ^*p*^p < 0.05- statistical significance versus 6-OHDA lesioned striatum of PRE-084-treated mice (Kruskal–Wallis test, Dunn’s post hoc test).

**Figure 2:**
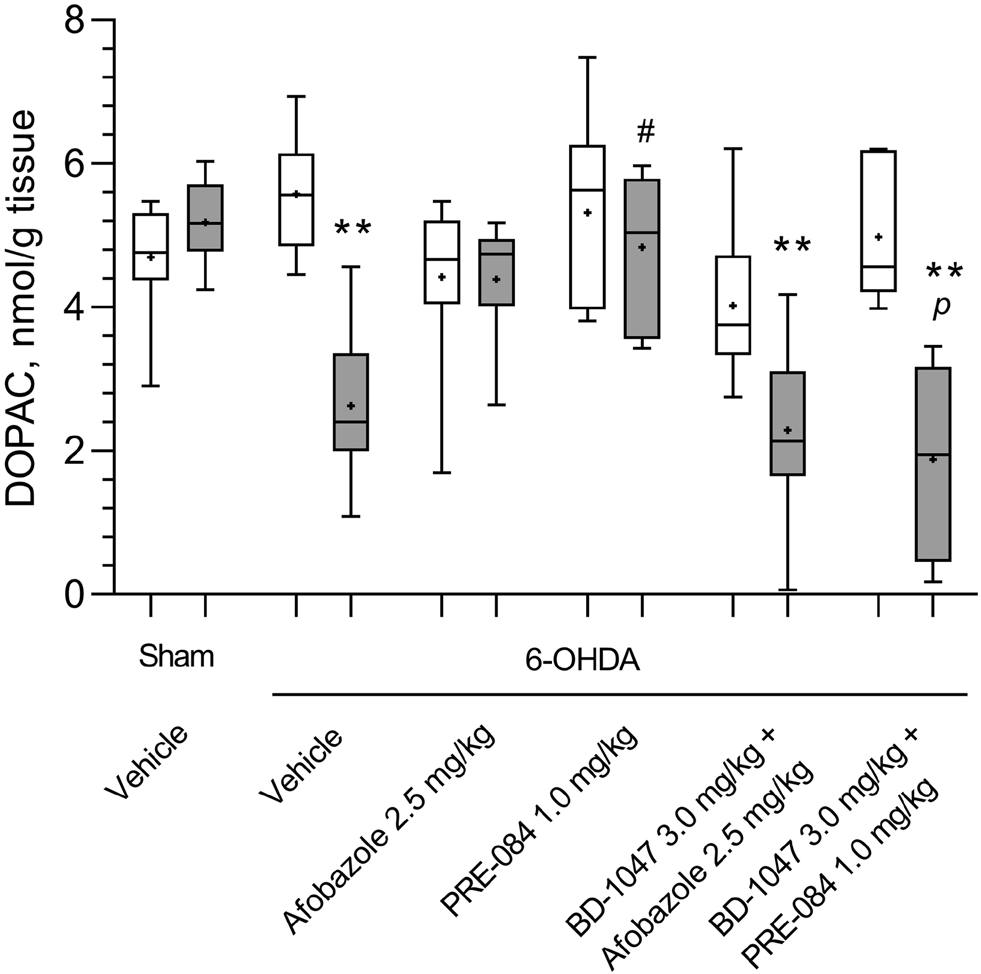
The influence of Sigma1R ligand administration over 14 days on dihydroxyphenylacetic acid content in the intact and 6-OHDA-lesioned striatum of ICR mice. Data are presented as the Mdn (min-max). “+” – the mean. Sham – sham-operated mice. 6-OHDA – 6-OHDA-lesioned mice. Experimental groups consisted of 10 mice except for afobazole-treated only, which was 9. A significant difference between the contra- and ipsilateral striata was observed in animals with 6-OHDA lesions that received vehicle or a combination of BD-1047 with afobazole or PRE-084 (p < 0.01, Wilcoxon test).  - contralateral striatum,  - ipsilateral striatum. **p < 0.01 – statistical significance versus lesioned striatum of sham-operated mice (Kruskal–Wallis test, Dunn’s post hoc test). ^#^p < 0.05 – statistical significance versus 6-OHDA lesioned striatum of vehicle treated mice (Kruskal–Wallis test, Dunn’s post hoc test). ^*p*^p < 0.05- statistical significance versus 6-OHDA lesioned striatum of PRE-084-treated mice (Kruskal–Wallis test, Dunn’s post hoc test).

We found that the DA content in the 6-OHDA lesioned striatum of vehicle-treated mice decreased two-fold compared to that in the contralateral striatum and in the damaged striatum of sham-operated mice (Fig. 1). Treatment with afobazole at 2.5 mg/kg for 14 days starting 30 minutes after the surgery markedly mitigated neurotoxic action, bringing DA content to the level observed in the damaged striatum of sham-operated mice (Fig. 1). The Sigma1R agonist PRE-084 administered at 1.0 mg/kg had a similar effect to that of afobazole (Fig. 1). The pre-administration of the selective Sigma1R antagonist BD-1047 at a dose of 3.0 mg/kg abolished the action of afobazole and PRE-084. The vulnerability of the PRE-084 effect was of higher significance (Fig. 1). The DOPAC level was decreased in the 6-OHDA lesioned striatum in vehicle-treated mice compared to that in the contralateral striatum and in the damaged striatum of sham-operated mice and was correlated with decreased DA content (Fig. 2). Although DA concentration increased in response to afobazole treatment, DOPAC content was in an intermediate range between concentrations observed in sham-operated and 6-OHDA-lesioned mice (Fig. 2). The administration of the Sigma1R agonist PRE-084 markedly increased DOPAC content compared to that in the 6-OHDA lesioned mice and restored DA content to the value observed in the control group (Fig. 2). In contrast, the Sigma1R antagonist BD-1047 administered daily 30 minutes prior to afobazole or PRE-084 diminished DOPAC content to the level of that in 6-OHDA lesioned vehicle-treated mice. BD-1047 markedly counteracted the action of PRE-084 (Fig. 2).

**Figure 3:**
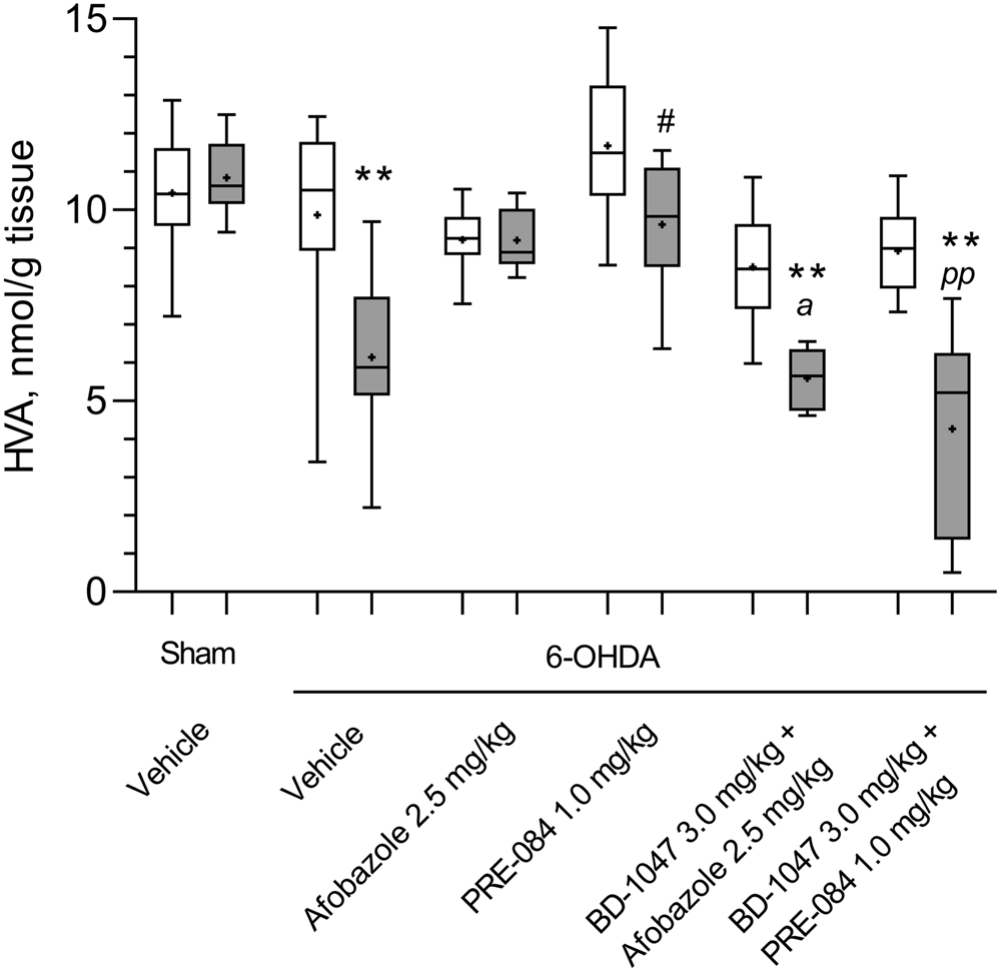
The influence of Sigma1R ligand administration over 14 days on homovanillic acid content in the intact and 6-OHDA-lesioned striatum of ICR mice. Data are presented as the Mdn (min-max). “+” – the mean. Sham – sham-operated mice. 6-OHDA – 6-OHDA-lesioned mice. Experimental groups consisted of 10 mice except for afobazole-treated only, which was 9. A significant difference between the contra- and ipsilateral striata was observed in animals with 6-OHDA lesions that received vehicle and PRE-084 (p < 0.05, Wilcoxon test), a combination of BD-1047 with afobazole or PRE-084 (p < 0.01, Wilcoxon test).  -contralateral striatum,  - ipsilateral striatum. **p < 0.01 - statistical significance versus lesioned striatum of sham-operated mice (Kruskal–Wallis test, Dunn’s post hoc test). #p < 0.05 - statistical significance versus 6-OHDA lesioned striatum of vehicle-treated mice (Kruskal–Wallis test, Dunn’s post hoc test). ^*a*^p < 0.05- statistical significance versus 6-OHDA lesioned striatum of afobazole-treated mice (Kruskal–Wallis test, Dunn’s post hoc test). ^*pp*^p < 0.01- statistical significance versus 6-OHDA lesioned striatum of PRE-084-treated mice (Kruskal–Wallis test, Dunn’s post hoc test).

Similar to the DOPAC content, the content of HVA was also reduced in the lesioned striatum of vehicle-treated mice, and afobazole had no significant effect on this value (Fig. 3). The HVA content in the 6-OHDA-lesioned striatum of PRE-084-treated mice was lower than that in the intact striatum but was markedly higher than that in the lesioned striatum of vehicle-treated mice. The pre-administration of the Sigma1R antagonist BD-1047 affects afobazole- and PRE-084-treated mice, preventing an increase in HVA (Fig. 3). No significant differences in DA, DOPAC and HVA contents were detected in intact striatum between experimental groups (Figs 1–3). The metabolic ratios DOPAC/DA, HVA/DA and (DOPAC + HVA)/DA were significantly elevated in the 6-OHDA lesioned striatum of vehicle-treated mice. Afobazole treatment lowered the HVA/DA and (DOPAC + HVA)/DA ratios, whereas PRE-084 had no detectable effect (Table 1).

**Table 1:** The influence of Sigma1R ligand administration over 14 days on dopamine turnover in intact and 6-OHDA- lesioned striatum of ICR mice.

Experimental groups		DOPAC/DA	HVA/DA	(DOPAC + HVA)/DA	
Sham	Vehicle	C n = 10	0.06 (0.06–0.07)	0.14 (0.13–0.15)	0.2 (0.19–0.21)
		I n = 10	0.07 (0.06–0.07)	0.14 (0.13–0.15)	0.2 (0.19–0.22)
6-OHDA	Vehicle	C n = 10	0.08 (0.07–0.09)	0.14 (0.11–0.16)	0.21 (0.19–0.24)
		I n = 10	0.1 (0.08–0.12) **p = 0.007 **^**p = 0.0195	0.23 (0.19–0.3) **p = 0.0006 **^^**p = 0.002	0.32 (0.27–0.42) **p = 0.0005 **^^**p = 0.002
	afobazole (2,5 mg/kg)	C n = 9	0.06 (0.05–0.07)	0.13 (0.11–0.14)	0.19 (0.16–0.2)
		I n = 9	0.07 (0.06–0.08)	0.14 (0.13–0.16) ^**#**^p = 0.014 ^^p = 0.0039	0.22 (0.2–0.23) ^**#**^p = 0.016 ^p = 0.027
	PRE-084 (1 mg/kg)	C n = 10	0.08 (0.06–0.08)	0.15 (0.14–0.17)	0.24 (0.2–0.25)
		I n = 10	0.08 (0.07–0.09)	0.16 (0.14–0.17)	0.24 (0.21–0.26)
	BD-1047 (3 mg/kg) + afobazole (2,5 mg/kg)	C n = 10	0.05 (0.05–0.06)	0.11 (0.11–0.13) ^**#**^p = 0.044 **^^**p = 0.0059	0.16 (0.16–0.18) **^**p = 0.037
		I n = 10	0.06 (0.04–0.09) ^#^p = 0.019	0.14 (0.13–0.24)	0.22 (0.18–0.33) ^#^p = 0.028
	BD-1047 (3 mg/kg) + PRE-084 (1 mg/kg)	C n = 10	0.07 (0.07–0.08)	0.13 (0.12–0.14)	0.2 (0.19–0.21)
		I n = 10	0.06 (0.02–0.09) ^#^p = 0.046	0.15 (0.04–0.22) ^#^p = 0.033	0.22 (0.06–0.32) ^#^p = 0.026

Data are presented as the Mdn (q25–75). Sham – sham-operated mice. 6-OHDA – 6-OHDA-lesioned mice. C- contralateral (intact) striatum, I – ipsilateral (lesioned) striatum

Kruskal–Wallis test, Dunn’s post hoc test:

**p < 0.01 - statistical significance versus lesioned striatum of sham-operated mice.

^#^p < 0,05 - statistical significance versus 6-OHDA lesioned striatum of vehicle treated mice.

Wilcoxon test:

^p < 0.05, ^^p < 0.01- statistical significance versus contralateral striatum.

**Figure 4:**
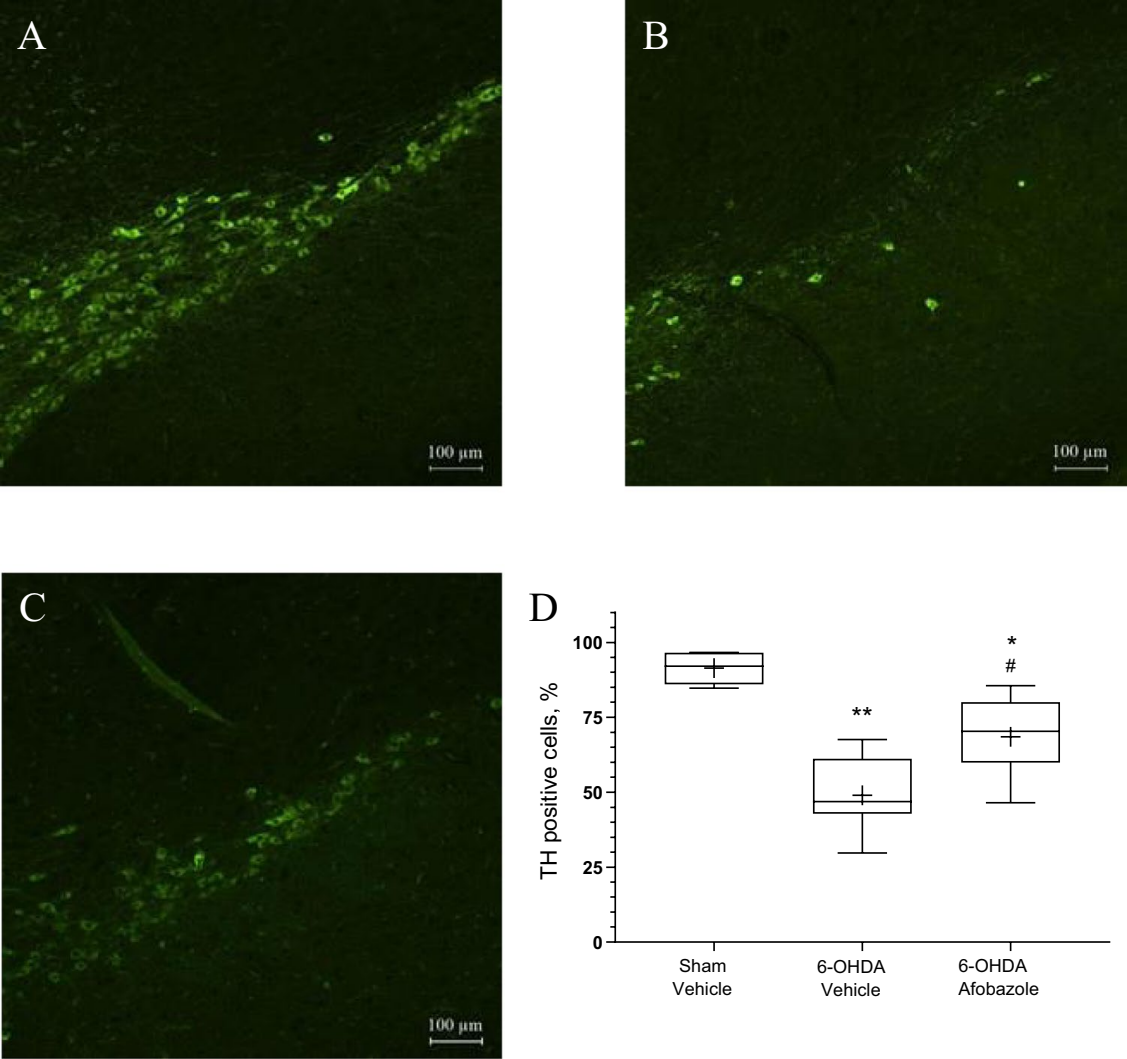
The influence of afobazole on the count of TH + neurons in the ipsilateral SNc in the model of Parkinson’s disease induced by a single injection of 6-OHDA. (**A**) Sham-operated mice (n = 6). (**B**) Mice with 6-OHDA lesions (n = 7). (**C**) Afobazole-treated mice with 6-OHDA lesions (n = 9). Imaging was carried out at 10x magnification. (**D**) TH-positive cell count. The count was performed in 3–6 slices of the SNc (rostral, medial and caudal regions) from each animal obtained under the same microscopy conditions: 40x magnification, exposition time 800 ms, and 1.1 gain. The density of SNc neurons on the ipsilateral side is expressed as a percentage of that on the contralateral side. Data are presented as the Mdn (min-max). “+” – the mean. Sham – sham-operated mice. 6-OHDA – 6-OHDA-lesioned mice. *p < 0.05, **p < 0.01- statistical significance versus sham-operated mice (ANOVA, Holm-Sidak post hoc test). ^#^p < 0.05 - statistical significance versus 6-OHDA lesioned mice treated with vehicle (ANOVA, Holm-Sidak post hoc test).

**Figure 5:**
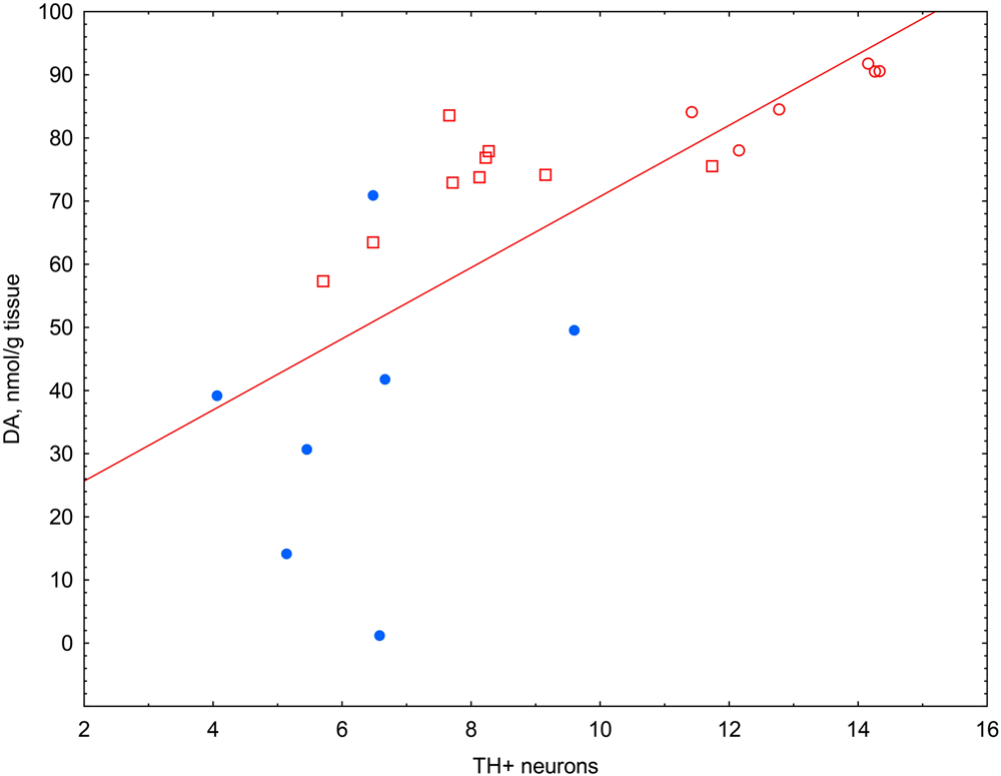
Correlation analysis of the TH+ neuron count in the ipsilateral SNc and DA content in the striatum. Light circle – sham-operated vehicle-treated animals (n = 6); dark circle – 6-OHDA-lesioned vehicle-treated animals (n = 7); light square – 6-OHDA-lesioned afobazole-treated animals at a dose of 2.5 mg/kg (n = 9); blue color – cluster 2, red color – cluster 1; R = 0.85 p = 0.000001; line of best fit equation DA = 14.395 + 5.6337*x.

Prolonged treatment with afobazole at a dose of 2.5 mg/kg preserved DA content in the 6-OHDA lesioned striatum, was accompanied by a marked increase in the relative number of TH+ neurons in the SNc (Fig. 4) and latency to fall in rotarod test (S. Fig. 1). A strong positive correlation was revealed between the DA level and the number of SNc TH+ neurons (R = 0.85, p < 0.00001) (Fig. 5).

**Figure 6:**
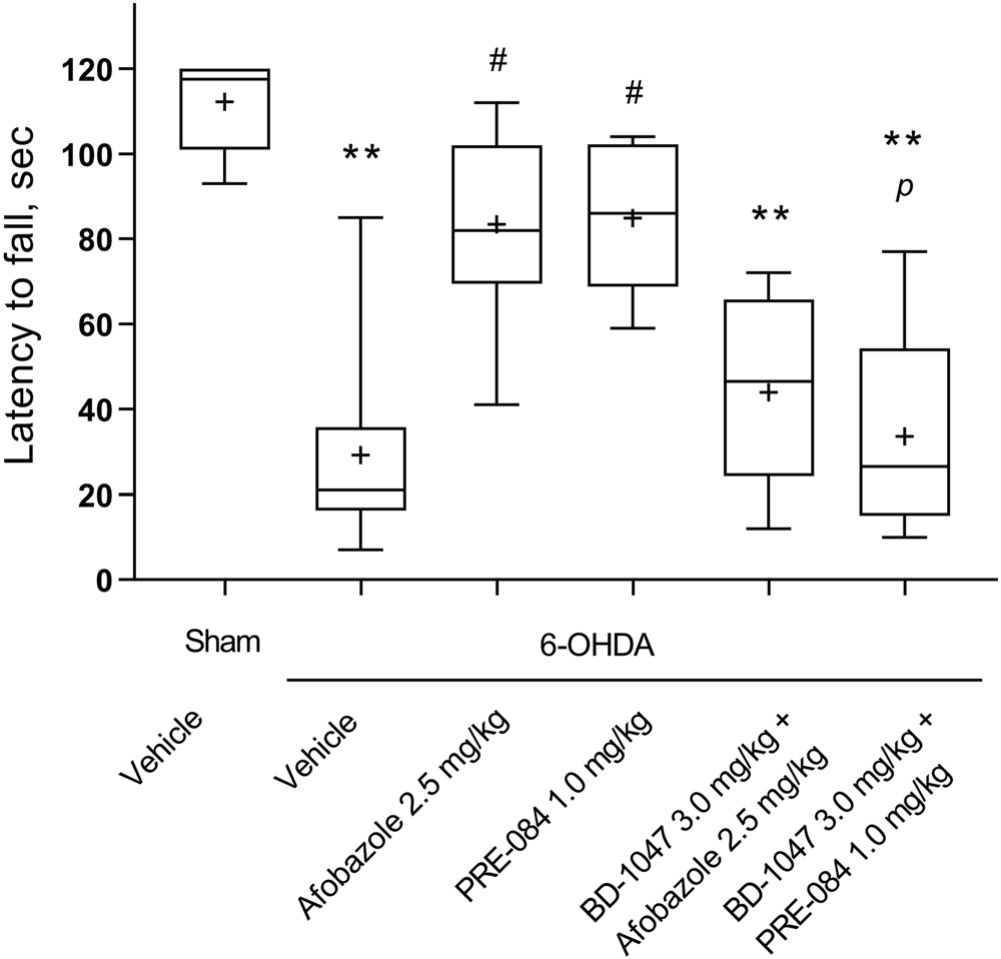
The influence of Sigma1R ligand administration over 14 days on latency to fall during a fixed-speed rotarod test in 6-OHDA-lesioned ICR mice. Data are presented as the Mdn (min-max). “+” – the mean. Sham – sham-operated mice. 6-OHDA – 6-OHDA-lesioned mice. Experimental groups consisted of 10 mice except for afobazole-treated only, which was 9. **p < 0.01- statistical significance versus sham-operated mice (Kruskal–Wallis test, Dunn’s post hoc test). #p < 0.05- statistical significance versus 6-OHDA-lesioned mice treated with vehicle (Kruskal–Wallis test, Dunn’s post hoc test). ^*p*^p < 0.05 - statistical significance versus 6-OHDA-lesioned mice treated with PRE-084 (Kruskal–Wallis test, Dunn’s post hoc test).

**Figure 7:**
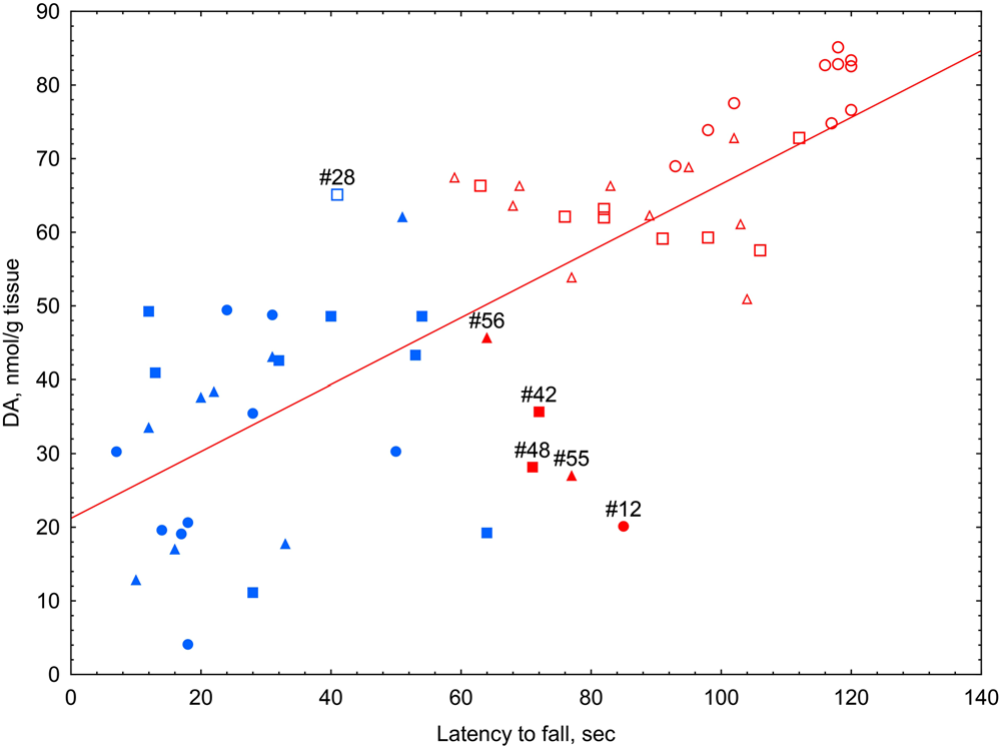
Correlation and cluster analysis of the association between striatal DA content and the latency to fall in the rotarod test. Light circle – sham-operated vehicle-treated animals; dark circle – 6-OHDA-lesioned vehicle-treated animals; light square – 6-OHDA-lesioned afobazole-treated animals at a dose of 2.5 mg/kg; dark square – 6-OHDA-lesioned afobazole-treated animals at a dose of 2.5 mg/kg with BD-1047 pretreatment at a dose of 3.0 mg/kg; light triangle – 6-OHDA-lesioned PRE-084-treated animals at a dose of 1 mg/kg; dark triangle – 6-OHDA-lesioned PRE-084- treated animals at a dose of 1.0 mg/kg with BD-1047 pretreatment at a dose of 3.0 mg/kg; blue color – cluster 2, red color – cluster 1. K-means data clustering demonstrated that the first cluster consisted of sham-operated and afobazole- or PRE-084-treated mice (except case #28). The second cluster consisted of vehicle-treated or BD-1047 pretreated mice (except cases #12, #42, #48, #55, and #56). R = 0,77 p = 0,000000; line of best fit equation DA = 21,2193 + 0,4532*x. Experimental groups consisted of 10 mice except for afobazole-treated only, which was 9.

In the rotarod test, the median latency to fall of vehicle-treated mice with 6-OHDA lesions decreased five-fold compared to that of sham-operated animals (Fig. 6). Afobazole and PRE-084 significantly increased the latency to fall in hemiparkinsonic animals. The pre-administration of BD-1047 antagonized afobazole action. Similarly, pretreatment with BD-1047 decreased the latency to fall of PRE-084-treated mice compared to that of sham-operated mice or mice treated with PRE-084 (Fig. 6). No significant difference in the latency to fall in the rotarod test was observed in naive intact and vehicle-treated animals versus sham-operated mice (S. Fig. 2). The ligands of the Sigma1R and its combinations did not affect the performance of naive mice in the rotarod test (S. Fig. 2). Our data show a strong positive correlation between DA content in the striatum and latency to fall (R = 0.77, p < 0.00001) (Fig. 7).

K-means data clustering demonstrated the partitioning of experimental mice into two major groups. The first cluster was represented by sham-operated and afobazole- or PRE-084-treated mice (except case #28), while the second cluster consisted of vehicle-treated or BD-1047-pretreated mice (except cases #12, #42, #48, #55, and #56). Animals whose experimental characteristics stood out from those of the group and turned out to be in the opposite cluster were at the maximum distance from the center of their cluster (Fig. 7).

## Discussion

The current study, for the first time, characterizes the contribution of Sigma1Rs in the neuroprotective effects of afobazole in an *in vivo* 6-OHDA model of Parkinson’s disease. The unilateral intrastriatal injection of 6-OHDA leads to a decrease in the DA content in the striatum, which is cross-correlated with the death of TH+ neurons in the SNc and low latency to fall in the rotarod test. These phenotypic characteristics represent pathogenetic mechanisms of Parkinson’s disease and are consistent with previously reported results in this model^44,45^. Afobazole administered at a dose of 2.5 mg/kg prevents the development of disorders caused by neurotoxicity, therefore demonstrating its neuroprotective activity proven by the high cross-correlation between DA content in the striatum and latency to fall in the rotarod test or TH+ count. Neuroprotection in the 6-OHDA model of Parkinson’s disease is characterized by the prevention of TH+ neuron death and the normalization of DA content in the striatum and movement activity^13,46,47^. A significant decrease in DA metabolic ratios caused by afobazole is also in line with experimental data that characterize neuroprotection in models of Parkinson’s disease^11,48^.

The effects of afobazole and the Sigma1R agonist PRE-084 administered at 1.0 mg/kg show many similarities, as confirmed by cluster analysis. One of the two clusters of experimental animals was represented by sham-operated mice and mice that received afobazole or PRE-084 only. The observed effect of afobazole is consistent with the previously described neuroprotective effect of PRE-084 in C57BL/6 mice in the 6-OHDA model of Parkinson’s disease^11^.

Pre-treatment with the selective Sigma1R antagonist BD-1047 abolished the restorative effect of afobazole on DA content, as well as the latency to fall in the rotarod test. These results suggest that Sigma1Rs are implicated in afobazole pharmacological activity. The clustering of vehicle-treated animals with animals that received BD-1047 pretreatment prior to afobazole and PRE-084 administration implies that BD-1047 interferes with afobazole and PRE-084 activity in a similar manner. The effects of afobazole on DA and DOPAC content and latency to fall in the rotarod test were less affected by BD-1047 pre-administration than PRE-084. These observations may argue for the existence of additional molecular mechanisms, in addition to Sigma1Rs, that mediate the neuroprotective effect of afobazole^38,49,50^. Thus, the lack of significant elevation in DOPAC content, along with the normalization of DA levels after afobazole treatment, suggests the inhibition of MAO-A by the drug^49^, which may facilitate the neuroprotective action of afobazole on neurons of the nigrostriatal pathway^51^. The results obtained in our study are consistent with previously published data on the cytoprotective and neuroprotective effects of afobazole, demonstrating the role of Sigma1Rs in the activation of antiparkinsonian mechanisms. 6-OHDA was shown to cause abundant Ca^2+^ uptake in neurons^52^. In models of acidosis and cortical neuron ischemia, the administration of afobazole substantially decreased Ca^2+^ uptake; however, the use of the selective Sigma1R antagonists BD-1047 and BD-1063 weakened this effect^53^. The activation of microglia is an important component of Parkinson’s disease pathogenesis; in fact, microglia activation was observed after 6-OHDA injection, suggesting the relevance of this animal model^54,55^. Injured neurons release ATP and UTP, which serve as signaling molecules mediating microglial activation. Afobazole blocked the migration of microglia into the region with increased concentrations of ATP and UTP *in vitro*, while selective Sigma1R antagonists prevented this effect^56^. Moreover, the Sigma1R antagonist BD-1047 also decreased the suppressive effect of afobazole on microglial activation by Aβ_25-35_^57^. In our previous studies, the effects of afobazole were ablated by a sigma-2 receptor antagonist^56,57^. Afobazole does not interact with the sigma-2 receptor, as demonstrated by a binding assay^38^. Sigma1Rs and sigma-2 receptors are different proteins encoded by diverse genes^9,58,59^. The interconnection of these two proteins in physiological processes is poorly understood^60–62^. However, the sigma-2 receptor may be a part of the protective pathway that is triggered by the interaction of the Sigma1R chaperone with afobazole. The development of Parkinson’s disease and other neurodegenerative conditions is accompanied by impaired protein folding and the presence of protein aggregates followed by neuronal dystrophy. The main ER chaperone BiP is found in complex with Sigma1R. Activation induced by Sigma1R agonists causes the dissociation of that complex and the activation of BiP^14,63,64^. There is evidence that Sigma1R contributes to the regulation of the proteasomal degradation of aberrant proteins, amyloid in particular^65,66^. It is known that amyloid proteins can accumulate within cells, causing neurotoxicity^67,68^. Afobazole increases the survival of cortical neurons incubated with amyloid Aβ_25-35_ by inhibiting excessive Ca^2+^ uptake and NO production. The selective Sigma1R antagonist BD-1047 also prevented this action of afobazole^57^. The development of Parkinson’s disease is accompanied by the increased production of quinone compounds, such as dopamine quinone^69,70^, as well as by oxidative stress^71,72^. The contribution of Sigma1Rs to the protective effect of afobazole in the model of menadione cytotoxicity was also previously reported by our group^39^.

Taken together, the current study demonstrates the important role of Sigma1Rs in the neuroprotective effect of afobazole in the 6-OHDA model of Parkinson’s disease and suggests that the use of Sigma1R agonists could be beneficial as a Parkinson’s disease treatment.

## Methods

### Chemicals

The following chemicals were used: afobazole (5‐ethoxy‐2‐[2‐(morpholino)‐ethylthio]benzimidazole dihydrochloride) (FSBI “Research State Zakusov Institute of Pharmacology”), PRE-084 (Tocris), BD-1047 (Tocris), 6-hydroxydopamine hydrochloride (6-OHDA), ascorbic acid, NaCl, sucrose, paraformaldehyde (PFA), polyclonal antibodies against tyrosine hydroxylase T8700 (Sigma-Aldrich), secondary antibodies conjugated with CF488 fluorochrome SAB4600045 (Sigma-Aldrich), FluoroShield, 4′,6-diamidino-2-phenylindole, Triton X-100, chloral hydrate, 3,4-dihydroxybenzylamine hydrobromide (DHBA), dopamine (DA), 3,4-dihydroxyphenylacetic acid (DOPAC), homovanillic acid (HVA), KH_2_PO_4_, H_3_PO_4_, HClO_4_, citric acid, EDTA-Na_2_, octanesulfonic acid, acetonitrile, and Tissue Tek O.C.T. medium.

### Experimental animals

The study was performed in male ICR (CD-1) mice (25–30 g, n = 152) obtained from Pushchino Breeding Center (Branch of the Institute of Bioorganic Chemistry, Russian Academy of Sciences). Animals were housed under standard vivarium conditions (20–22 °C, 30–70% humidity, 12-hour light/dark cycle) in plastic cages with sawdust bedding and 10 animals per cage.

### Ethical approval

All experimental procedures were approved by the bioethics committee of the FSBI “Research Zakusov Institute of Pharmacology”. All applicable national^73^ and international^74^ guidelines for the care and use of experimental animals were followed.

### 6-OHDA lesion

Thirty minutes prior to surgery, the animals were anesthetized with chloral hydrate (400 mg/kg intraperitoneally)^45,75^. Anesthetized animals were placed into the stereotaxic frame (Stoelting Co., United Kingdom), and 6-OHDA was injected into the right striatum according to the coordinates A = 0.4, L = 1.8, and V = -3.5 relative to bregma^45^. 6-OHDA was dissolved at 5 μg per 1 μL of a solution containing 0.9% NaCl and 0.02% ascorbic acid. The experimental animals were injected with 1 μL of the 6-OHDA solution at a rate of 0.5 μL/min using a Hamilton syringe equipped with a 30-gauge stainless steel needle. The needle was withdrawn 2 min after the injection. Sham-operated animals were injected with 1 μL of saline with 0.02% ascorbic acid at the same coordinates.

### Treatment with drugs

All drug substances were dissolved in water for injections immediately before administration and were intraperitoneally injected at a volume of 0.1 ml/10 g body weight. Afobazole at a dose of 2.5 mg/kg, PRE-084 at a dose of 1.0 mg/kg or vehicle were injected daily for 14 days, with course starting 30 minutes after surgery. The selective Sigma1R antagonist BD-1047, at a dose of 3.0 mg/kg, was injected 30 minutes prior to afobazole or PRE-084. The animals were divided as follows. Sham-operated vehicle-treated mice (n* = *10) and five groups with 6-OHDA lesions: mice treated with afobazole (n = 9), PRE-084 (n = 10), BD-1047 following the administration of afobazole (n = 10) or PRE-084 (n = 10), and vehicle-treated (n = 10). One animal with a 6-OHDA lesion that received afobazole was excluded because of death during surgery. To verify whether compounds used in the study affect motor behavior in rotarod test, 70 naive mice were divided into the following groups: intact mice (n = 10), animals that received vehicle (n = 10), afobazole (n = 10), PRE-084 (n = 10), BD-1047 (n = 10) and a combination of BD-1047 and afobazole (n = 10) or PRE-084 (n = 10). Additional 22 animals used for immunohistochemical assay were divided as follows: sham-operated vehicle-treated mice (n = 6) and two groups of mice with 6-OHDA lesions vehicle- (n = 7) and afobazole-treated (n = 9).

All animals were treated with the same doses of studied compounds for 14 days, except for naive intact animals.

### Rotarod test

Motor behavior was studied in male ICR mice utilizing a Rota-rod/RS LE 8500 apparatus (Panlab/Harvard apparatus). Two training sessions spaced 24 hours apart were performed to acclimate the experimental animals to the apparatus and to exclude hypodynamic animals from the study. The first training session was performed on the twelfth day after surgery. Each animal was placed on the rod twice as it rotated at a rate of 4 rpm, with a 1-hour interval between sessions. During the second session, the rotation speed was increased to 10 rpm. Animals unable to hold on to the rod for more than 1 minute were excluded from the study^44^. In the present study, all animals were able to hold on to the rod for more than one minute, so none of them were excluded from the experiment.

On the 14th day after surgery, mice were placed onto a rod rotating at 20 rpm, and latency to fall was measured. Each animal was tested three times, with 30-minute intervals between attempts. If the experimental animal was able to hold on to the rod for 120 s, the measurement of time was stopped. The maximum time of the three attempts was used for statistical analysis. On days with training sessions and the rotarod test, drugs were administered after pretraining or testing.

### HPLC-ED technique

Dopamine and its metabolites were measured in the damaged and intact striata of the mouse brain using the high-performance liquid chromatography technique with electrochemical detection (HPLC-ED). Fourteen days after the 6-OHDA administration, the mice were decapitated, and the brains were removed. The left and right striata were dissected on wet ice covered with filter paper dampened in 0.32 M sucrose solution at a temperature of 0–4 °C. Each striatum was frozen in liquid nitrogen (-196 °C), weighed, and stored at -80 °C. To measure the contents of dopamine and its metabolites, the striatum was homogenized in 0.1 M НClO_4_ in a TissueLyserLT bead homogenizer (Qiagen, Germany) at a frequency of 45 bit/min for 5 minutes. DHBA was added as an internal standard at a concentration of 0.25 nmol/mL. The samples were centrifuged at 10 000 *g* and 4 °C for 10 min. Twenty μL of the supernatant was applied onto a Kromasil C-18 4.6 × 150 analytical column (Dr. Maisch, Germany) using an SIL-20 ACHT autosampler (Shimadzu, Japan). Monoamines and their metabolites were separated in the column using 0.1 M citrate-phosphate buffer containing 0.3 mm sodium octanesulfonate, 0.1 mm EDTA, and 8% acetonitrile (pH 3.0) as the mobile phase. Determination of dopamine and its metabolites was performed using a Shimadzu LC-20 Prominence chromatographic station (Shimadzu, Japan) equipped with an ESA 5011 (E1 = –175; E2 = +250) electrochemical cell and a Coulochem III electrochemical detector (ESA, United States). The flow rate of the mobile phase was 1 mL/min. The results were processed on a PC using Multichrom 1.5 software (Ampersand, Russia). MAO and catechol-O-methyltransferase (COMT) are involved in the biodegradation of dopamine. Therefore, we used the metabolic ratio DOPAC/DA, which reflects MAO activity; HVA/DA, which reflects the COMT activity; and the total ratio (DOPAC + HVA)/DA^76^.

### Immunohistochemical analysis

Fourteen days after the surgery, the mice were decapitated, and the brains were extracted. Specimens were fixed for 24 hours by immersion in 4% PFA solution, soaked in 30% sucrose and embedded in Tissue Teck O.S.T. medium. Twelve micrometer thick serial frontal sections of the midbrain in the region of the SNc were prepared utilizing a Sakura Tissue Tec Cryo3 cryotome. Prior to staining, heat epitope retrieval was performed in 0.1 M citric buffer solution (pH 6.0) in a microwave oven in repeated cycles (600 W, 5 minutes). A large water bath that contained vials with slides and a mercury thermometer was placed into a microwave oven. Heating was intermitted so the temperature stayed at 93–96 °C. After cooling, the slides were rinsed with PBS and left for 1 hour in PBS containing 0.1% Triton X-100. To reveal dopamine-producing neurons in the SNc, slides were incubated with rabbit polyclonal antibodies against tyrosine hydroxylase (1:500 dilution) for 16 hours. Bound rabbit immunoglobulins were visualized by goat antibodies conjugated with CF488 fluorochrome. Primary and secondary antibodies were incubated at room temperature in a light-proof dampening chamber. Nuclei were counterstained with DAPI. All slides were coverslipped in Fluoroshield mounting medium. Imaging was carried out using a Nikon Eclipse fluorescence microscope equipped with a Nikon DS-Qi camera.

The density of TH-positive (TH+) neurons with nuclei in the slice plane was counted at 40x magnification using ImageJ software (National Institute of Health). The count was performed in 3–6 slices of the SNc (rostral, medial and caudal regions) from each animal obtained under the same microscopy conditions, i.e., exposition time 800 ms, gain 1.1. In each animal, cells from the ipsi- and contralateral sides with respect to 6-OHDA injection were counted in 5–7 fields of view (0.044 mm^2^), and the mean was calculated. The density of SNc neurons on the ipsilateral side was expressed as a percentage of the contralateral side.

### Statistical analysis

To evaluate the type of experimental data distribution, D’Agostino-Pearson and Shapiro-Wilk tests were used. Because the data for DA content and latency to fall in the rotarod test did not fit a Gaussian distribution in the majority of groups, the Wilcoxon test and Kruskal-Wallis test with Dunn’s post hoc test were used. ANOVA with the Holm-Sidak post hoc test was used to evaluate the statistical significance of the TH+ neuron count. Linear coupling analysis of DA content in the striatum with the relative TH+ neuron count or latency to fall in the rotarod test was performed using Spearman correlation analysis. Tabular data are presented as medians with lower and upper quartiles (Mdn (q25-75)). Data in histograms are presented as the median with the minimum and maximum (Mdn (min-max)) and the mean. Animals were classified using k-means clustering by DA content in the striatum and latency to fall in the rotarod test as variables. The population was divided into two clusters. Statistical analysis of raw data and the visualization of obtained results were performed using GraphPad Prism version 8.0.1 for Windows, GraphPad Software, La Jolla California USA, www.graphpad.com and Statistica (StatSoft, Inc).

## Supplementary information

Supplementary materials Chaperone Sigma1R mediates the neuroprotective action of afobazole in the 6-OHDA model of Parkinson’s disease

## Data Availability

The datasets analyzed in this study are available from the corresponding author upon reasonable request.
