# Supplementary material for: Chaperone Sigma1R mediates the neuroprotective action of afobazole in the 6-OHDA model of Parkinson’s disease
Source: Sci Rep. 2019 Nov 19;9:17020. doi: 10.1038/s41598-019-53413-w (PMC6863824; doi:10.1038/s41598-019-53413-w)
Supplement: Supplementary file 1 — Supplementary materials Chaperone Sigma1R mediates the neuroprotective action of afobazole in the 6-OHDA model of Parkinson’s disease [file 41598_2019_53413_MOESM1_ESM.pdf]

Supplementary materials

Chaperone Sigma1R mediates the neuroprotective action of afobazole in the 6-OHDA model of Parkinson's disease.

M.V. Voronin[1], I.A. Kadnikov[1]*, D.N. Voronkov[2], S.B. Seredenin[1]

[1] Federal State Budgetary Institution "Research Zakusov Institute of Pharmacology", Department of Pharmacogenetics, Moscow, Baltiyskaya street 8, 125315, Russian Federation

[2] Research Center of Neurology, Laboratory of Functional Morphochemistry, Volokolamskoe Highway 80, Moscow, 125367, Russian Federation

* ikadnikov@gmail.com

Supplementary figure 1: The influence of afobazole administration over 14 days on latency to fall during a fixed-speed rotarod test in 6-OHDA-lesioned ICR mice.

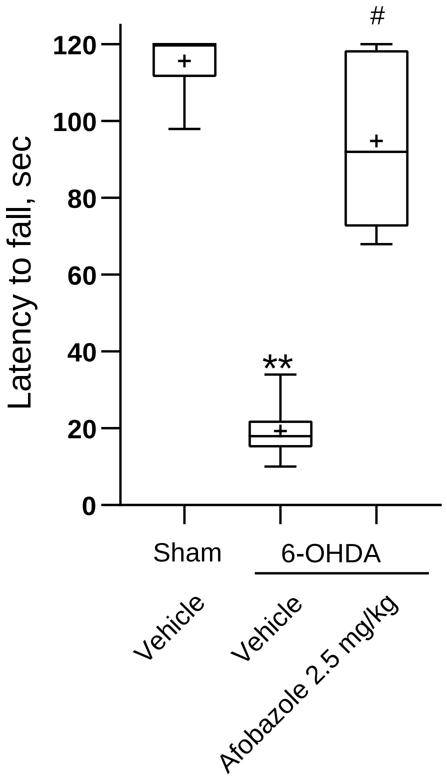

Data are presented as the Mdn (min-max). "+" – the mean. Sham – sham-operated vehicle-treated mice (n = 6). 6-OHDA – 6-OHDA-lesioned vehicle-treated mice (n = 7). Afobazole – 6-OHDA-lesioned animals that were administered afobazole at a dose of 2.5 mg/kg (n = 9).

** $p < 0.01$- statistical significance versus sham-operated mice (Kruskal–Wallis test, Dunn's post hoc test).

# $p < 0.05$- statistical significance versus 6-OHDA-lesioned mice treated with vehicle (Kruskal–Wallis test, Dunn's post hoc test).

Supplementary figure 2: The influence of Sigma1R ligand administration over 14 days on latency to fall in fixed speed rotarod of naive ICR mice.

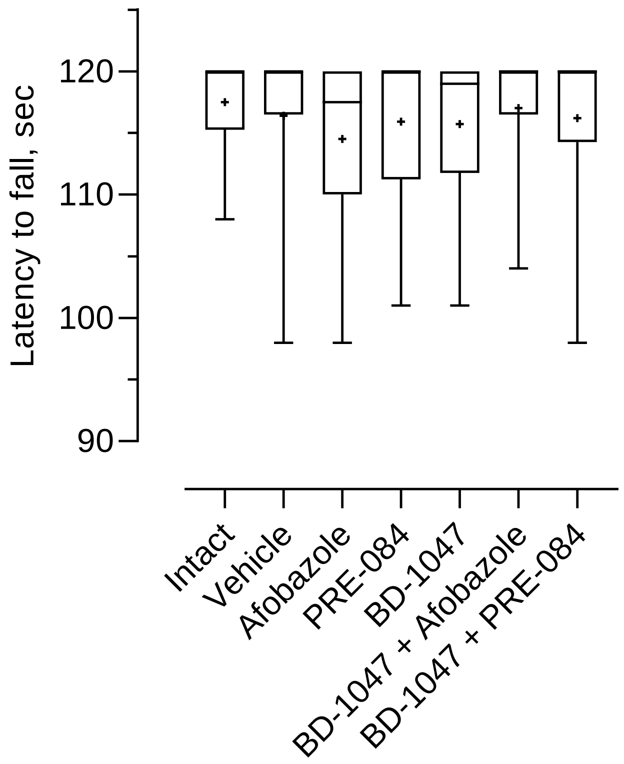

Data are presented as the Mdn (min-max). “+” – the mean. Experimental groups consisted of 10 animals each. Sham – sham-operated mice. Intact - untreated animals. Vehicle - vehicle (water for injections)-treated animals. Afobazole – animals that were administered afobazole at a dose of 2.5 mg/kg. PRE-084 – animals that were administered afobazole at a dose of 1.0 mg/kg. BD-1047 - animals that were administered BD-1047 at a dose of 3.0 mg/kg. BD-1047 + Afobazole - animals that were administered a combination of BD-1047 and afobazole at doses of 3.0 and 2.5 mg/kg, respectively. BD-1047 + PRE-084 - animals that were administered a combination of BD-1047 and afobazole at doses of 3.0 and 1.0 mg/kg, respectively.
